# Timescales and drivers of chlorophyll variability in a subtropical, long residence time estuary (Baffin Bay, Texas, USA)

**DOI:** 10.1371/journal.pone.0322053

**Published:** 2025-05-09

**Authors:** Emily K. Cira, Laura Beecraft, Michael S. Wetz

**Affiliations:** Harte Research Institute, Texas A&M University-Corpus Christi, Corpus Christi, Texas, United States of America; Gujarat Institute of Desert Ecology, INDIA

## Abstract

Little is known about the role of short-term (hourly to daily) processes on estuarine phytoplankton dynamics, though these can be important timescales of physical-chemical variability in coastal systems. This study coupled high-frequency (daily; 2015–2016) and low frequency (monthly; 2014–2018) sampling to quantify timescales of chlorophyll variability and potential drivers of phytoplankton blooms in a subtropical, long residence time estuary (Baffin Bay, Texas, USA). The dominant timescale of chlorophyll variability in the system was sub-monthly, which accounted for ~ 37% of variability, followed by interannual at ~ 30% and seasonal at 21%. From bloom events identified in the high frequency dataset, wind was a predominant factor related to short-term (daily resolution) blooms, often positively correlated with chlorophyll concentrations. Results expand upon the currently limited knowledge of short-term chlorophyll variability in subtropical, long residence time estuaries. Additionally, findings also offer insight into design considerations for sampling programs in similar systems.

## Introduction

Estuaries are dynamic systems, defined by dominant modes of environmental variability that range from hourly (tidal cycles) to seasonal (temperature and rainfall) to interannual (climate oscillations that also affect temperature and freshwater inflow). While variability on short-term (hourly to daily) timescales can be an important component of phytoplankton biomass variability in many coastal systems, it is often not captured with traditional monitoring frequencies [1–4]. Furthermore, few studies have quantified drivers of short-term variability in phytoplankton biomass, limiting our ability to understand phytoplankton dynamics, and their environmental drivers, in coastal systems [5,6].

Understanding of estuarine phytoplankton dynamics is further complicated by differences in timescales of phytoplankton variability among estuaries worldwide [2,7]. Low-latitude systems tend to have less seasonal variability in light and temperature than temperate systems, and as a result, phytoplankton seasonal patterns in low-latitude systems are generally depressed compared to temperate systems [7,8]. This suggests that generalizations about predominant drivers and scales of phytoplankton dynamics may not be transferable across estuaries occupying different latitudinal bands or climatic regimes. Tropical and subtropical systems have generally received little research interest to date [9], limiting our understanding of phytoplankton dynamics in them.

The goal of this study was to characterize timescales of chlorophyll variability in a subtropical, long residence time estuary (Baffin Bay, Texas, USA). In addition, relevant environmental data were analyzed to assess drivers of short-term variability in chlorophyll. Results improve our understanding of timescales of phytoplankton variability in the estuarine environment and contribute to a better understanding of phytoplankton population dynamics in the system. Additionally, by simulating various monitoring frequencies, we were able to demonstrate the effects of lower sampling frequency on the characterization of phytoplankton population dynamics.

## Methods

### Study site: Baffin Bay, Texas (USA)

Baffin Bay is a shallow (average depth ∼1–2 m) estuary located in a semi-arid region of the South Texas coast. It is fed by three small streams, two of which (Los Olmos and Petronila Creeks) often exhibit minimal flows except during high rainfall periods that are linked to El Niño conditions and episodic tropical systems (unpubl. United States Geological Survey streamflow data). The third stream (San Fernando Creek) exhibits continuous flow due to it receiving treated municipal wastewater discharge from 11 facilities. The relatively low inflow to Baffin Bay coupled with high evaporation rates in the region leads to frequent hypersaline conditions. Winds can be relatively strong in the region and are thought to play a dominant role in hydrography of the system [10]. Wind direction is predominantly from the southeast from May-September, driving water to the north in Laguna Madre. In contrast, it is not uncommon to have winds out of the north in winter, which can alter the flow regime considerably [10]. The nearest inlets that allow for exchange between the Laguna Madre and the Gulf of Mexico are Packery Channel (~41 km north of Baffin Bay), Aransas Pass (~70 km north of Baffin Bay), and Port Mansfield (~80 km south of Baffin Bay). These distances, along with diurnal tidal ranges of only ~2–3 cm, results in minimal overall tidal influence on the system. In fact, the dominant tidal signature is that of a long period, semiannual tide that results in changes in water level of ~ 50 cm. Additional water level change (10–20 cm) is driven by wind stress.

### Assessing timescales of chlorophyll variability

**Fig 1:**
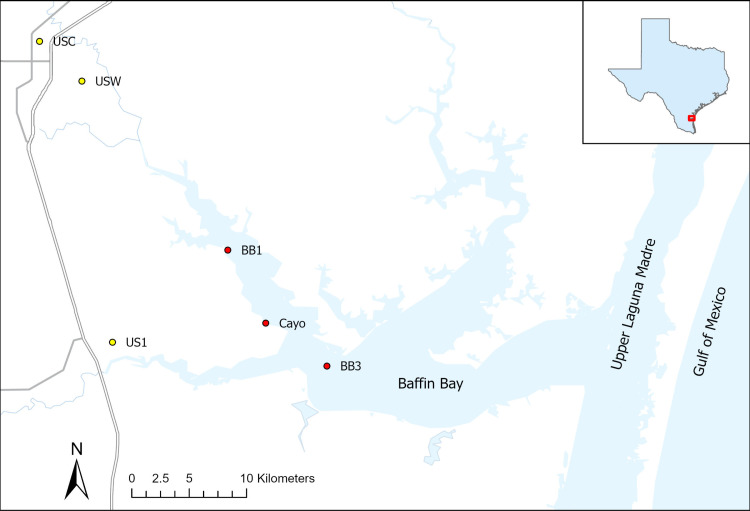
Map of Baffin Bay, Texas. Red circles indicate Baffin Bay monthly water quality sampling stations (BB1, BB3) or sonde deployment sites (Cayo, BB3). Yellow circles indicate stations where wind data and rainfall data were utilized, with labels taken from the first three letters of the station names (refer to Methods for full station IDs).

Data from a monthly monitoring program (2014–2018) was used to provide a first-order assessment of timescales of chlorophyll variability. Surface water (~0.1 m depth) was collected at a central bay site (BB3, Fig 1). Water samples were collected in 1 L acid-washed and deionized water-rinsed amber high-density polyethylene (HDPE) bottles attached to an extendable sampling stick. After collection, samples were stored in a cooler filled with ice and immediately transported back to the lab for filtration and further laboratory analysis. After homogenization, water samples were filtered through 0.7 µm pore size GF/F filters. Filters were stored frozen (-20 ^°^C) in sealed Vacutainers for analysis of chlorophyll *a*. Chlorophyll *a* was extracted from the filters by soaking for 18–24 hours in 90% HPLC-grade acetone at -20 ^°^C, after which chlorophyll *a* was determined fluorometrically with a Turner Trilogy fluorometer without acidification.

Chlorophyll variability was assessed with a multiplicative decomposition model using the decompTS function from the wql package in R [11]. This function partitions variability from the overall mean into annual, monthly, and sub-monthly (residual) components. The annual and monthly components consist of a coefficient for each year or month, representing the deviation of an individual year or month from the overall mean. The sub-monthly component captures variability not attributed to the annual or monthly components. The relative magnitude of annual and monthly coefficients was used to assess annual and seasonal trends (2014–2018). Additionally, output from this function was used to compare the relative magnitude of annual, monthly and sub-monthly variability (2014–2018), expressed as the standard deviation of each timescale component [2,12].

### High temporal resolution sampling program, 2015–2016

Water temperature, salinity, and chlorophyll (as *in vivo* fluorescence) were monitored at 15-minute intervals using Hydrolab DS5X sondes moored to pilings at ~ 0.5 m depth at two sites in Baffin Bay (BB3 and Cayo, Fig 1). Sondes underwent rigorous pre- and post-deployment calibration, and deployment times were limited to reduce potential fouling impacts on data collection. In addition, sondes were wrapped in copper foil to reduce fouling. Data collected at 15-minute intervals were subsequently converted to daily averages to remove diel signatures. Missing datapoints in the 15-minute interval data record were filled by linear interpolation prior to taking the daily average, and a daily average was omitted if more than one consecutive hour of data was missing within that 24-hour period. Gaps in data represent days when conductivity and/or chlorophyll sensors were not functioning or did not pass appropriate post-deployment checks, according to manufacturer’s recommendations. The chlorophyll probe was calibrated with rhodamine dye, and was analyzed as reported by the sonde, in µg·L ^-1^.

Bloom events were identified as chlorophyll increases of 50 µg·L^-1^ within a four-day period, which represents an approximate doubling of the mean chlorophyll concentration during the entire time series for both sites. When a bloom event was identified, sonde data from up to 14 days before and 14 days after the bloom were used to establish correlations between chlorophyll and environmental variables. Multiple blooms occurring within a 14-day period were combined and analyzed as one bloom event. Statistical analyses were conducted on bloom events that coincided with continuous coverage of water temperature (from sondes), wind (from Kingsville Naval Air Station, Station USW00012928; Fig 1) and rain (averaged from three sites in the Kingsville area: US1TXKL0011, USC00414810, USW00012928; Fig 1; retrieved from the National Climatic Data Center), resulting in six blooms that were used for analysis. Wind data were assessed as average daily wind speed (m·s^-1^) and the maximum daily wind speed (maximum wind speed sustained for 2-minute period, m·s^-1^).

To identify explanatory variables for chlorophyll during each bloom event, additive linear models were explored using the dredge function (MuMIn package [13]). The potential need for incorporation of one-day time-lagged variables was assessed with cross correlation functions. Models were compared with AICc (Corrected Akaike Information Criterion; MuMIn package [13]) and the quality of models was assessed through diagnostic plots showing normality and model fit. Models were assessed to remove collinear variables and were simplified using backwards regression to remove non-significant terms and create a significant model (α = 0.05). Note that rain did not occur during one bloom event (BB3 on 7/10/15–7/22/15), thus rain was not included as a variable in the analysis for that specific bloom event. Analyses were performed in R version 3.3.2 [14]. To supplement interpretation of bloom event analysis, monthly discrete sample data (temperature, salinity, ammonium [NH_4_^+^], nitrate + nitrite [N + N], silicate, orthophosphate, phytoplankton community composition) from two adjacent sites, BB1 and BB3 (Fig 1) were assessed (see methods in [15]).

### Effect of sampling frequency on chlorophyll estimates

**Fig 2:**
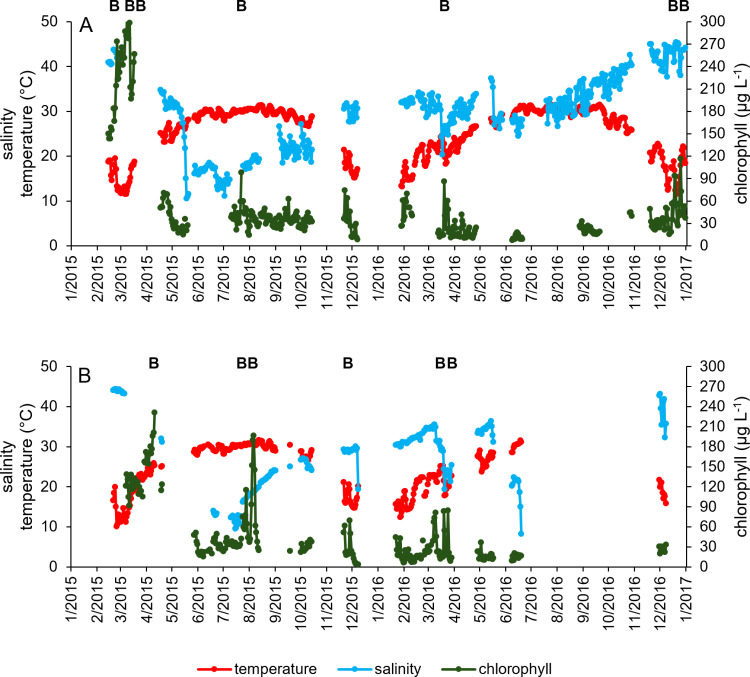
Daily averaged salinity, water temperature (°C) and chlorophyll (µg L^-1^) from continuous sonde deployments at two sites in Baffin Bay, A) BB3 and B) Cayo. Bloom events are indicated by a “B” along the top of each panel.

To determine the effects of different sampling frequencies on chlorophyll estimates, the 2015 daily sonde chlorophyll dataset from site BB3 was used as a basis for simulating the effects of two lower sampling frequencies (monthly, quarterly). The raw dataset had 171 days of useable chlorophyll data measured at irregular intervals, but here the data was treated as one “year” of data (i.e., one “year” = 171 days; S1 Fig; see also Fig 2A for the temporal distribution of the 171 chlorophyll datapoints in 2015). To mimic monthly or quarterly sampling of this “year” of data, the raw dataset was first divided into quarters (171/4 = 42.75; n of each quarter = 42 or 43) and months (171/12 = 14.25; n of each month = 14 or 15). Then, for each sampling frequency (quarterly and monthly), 1,000 random resampling simulations were conducted in which one random sample was collected for each quarter or month, resulting in 1,000 sets of 4 samples (quarterly sampling) or 12 samples (monthly sampling). Descriptive statistics (annual mean, minimum, maximum) were used to compare the resampled sets to the raw dataset.

## Results

### Timescales of chlorophyll variability (2014–2018)

**Fig 3:**
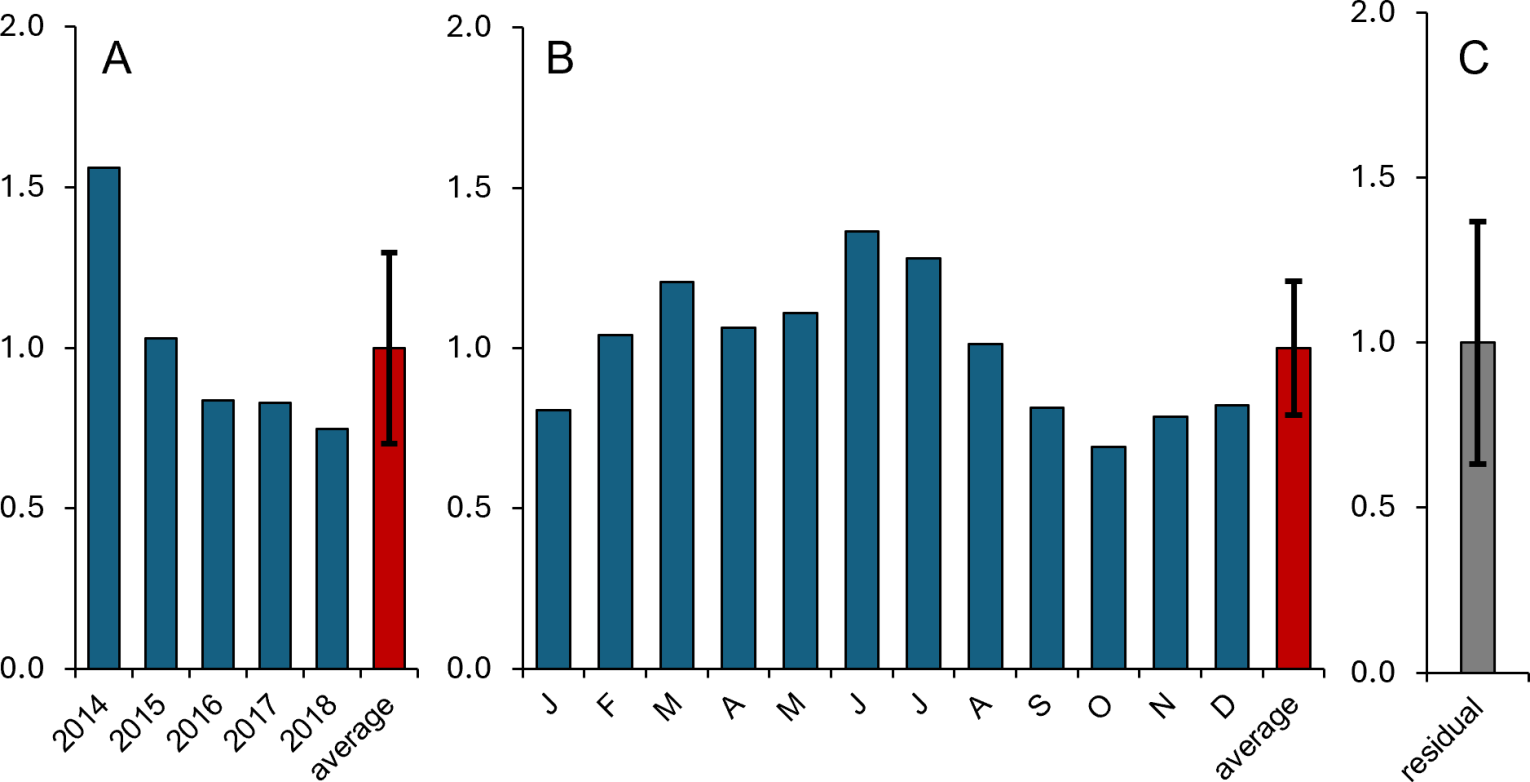
Annual (A), monthly (B), and residual (C) coefficients for chlorophyll from the decomposition model. For annual and monthly coefficients, individual coefficients are in blue, and the average is in red. The average residual (sub-monthly) coefficient is in gray. Error bars represent standard deviation for the average annual, monthly, and residual coefficients.

The decomposition model was used to assess the monthly monitoring program data. From the decomposition model output, there was an overall decrease in annual chlorophyll coefficients from 2014 to 2018 (Fig 3A). Seasonal trends are visible, with a chlorophyll peak in June – July (Fig 3B). The standard deviations of the decomposition model coefficients are representative of the variability contained in each model component (annual, seasonal, sub-monthly; error bars in Fig 3). The standard deviation of the seasonal component (0.21) was less than that of the annual component (0.30), indicating the magnitude of interannual variability was larger than that of seasonal variability during the 5-year sample period (2014–2018; Fig 3A and 3B). The standard deviation of the residual component (0.37) reflects variability not captured in the annual and monthly variation and likely relates to short-term (sub-monthly) variability (Fig 3C). The standard deviation of this component was greater than the seasonal and the annual components, reflecting the relative importance of short-term variability in the chlorophyll dynamics in Baffin Bay from 2014 to 2018.

### Spatial and temporal distribution of chlorophyll and environmental factors (2015–2016)

At both high-frequency sampling sonde deployment sites, water temperature followed a consistent seasonal trend, peaking at just over 30 °C in ~June – August and decreasing to near 10 °C in ~December – February (Fig 2). Variability in temperature was higher in the November-March timeframe, with temperature shifts of 5–10 °C noted over short timescales (< 3 d), while from June-August during the warmer months, temperature remained nearly constant (Fig 2). No clear seasonal trend in salinity was observed (Fig 2). Instead, salinity was characterized by sharp decreases due to rain events at several points in the record (Fig 2), with a gradual increase noted from May 2015 to the end of the record. Salinities ranged from ~10–45 at both sites (Fig 2).

**Fig 4:**
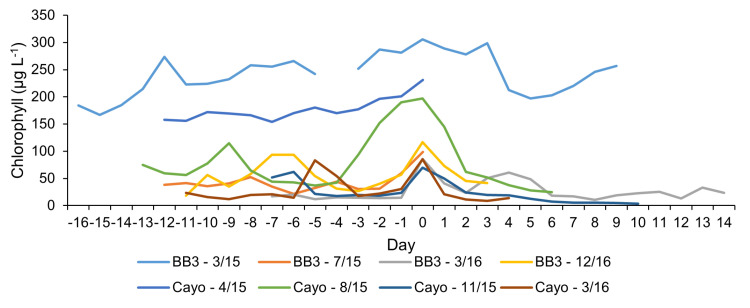
All identified bloom events by month/year of occurrence. Bloom events are plotted with the maximum chlorophyll concentrations (µg L^-1^) aligned with day 0 along the x- axis, with the extent of data coverage showing the chlorophyll concentrations on days leading up to, and after, the chlorophyll peak.

Chlorophyll concentrations did not exhibit clear seasonal trends. Concentrations peaked in March-April 2015 at both sonde deployment sites (BB3 and Cayo; Fig 2). Overall, concentrations were slightly higher and more variable at BB3 than Cayo (51.6 ± 61.3 µg L^-1^, 47.2 ± 47.9 µg L^-1^, respectively), though temporal coverage was also higher at BB3 (n = 309) than Cayo (n = 235). Bloom events were identified at both sites, across years and seasons (Figs 2 and 4). Blooms were often ephemeral, lasting < 3 days, though many bloom events also consisted of multiple chlorophyll peaks leading up to the day with maximum chlorophyll (day 0 on Fig 4).

### Potential drivers of blooms (2015–2016)

**Fig 5:**
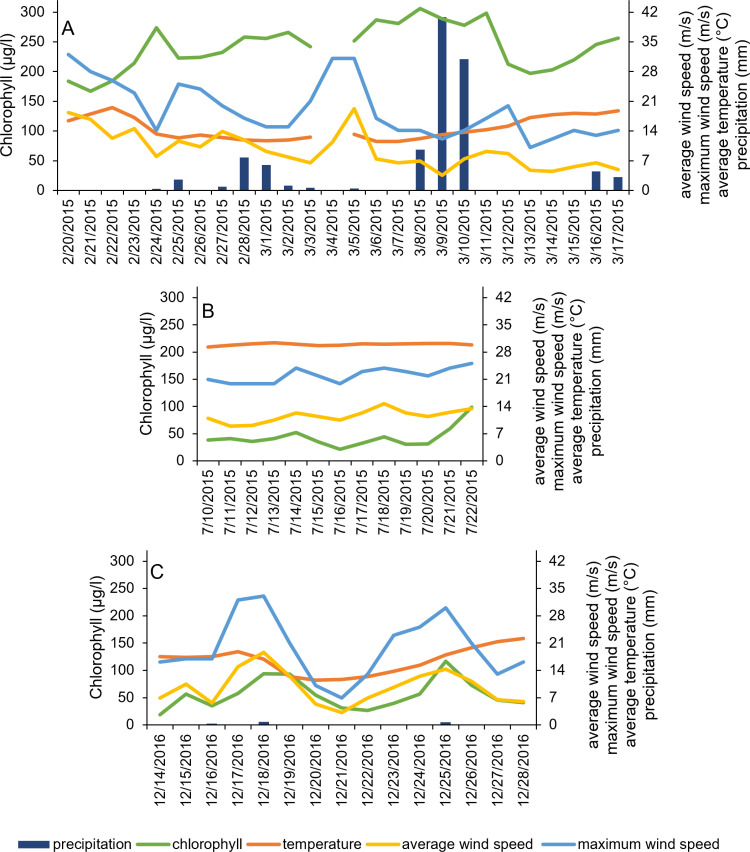
Chlorophyll (µg L^-1^), temperature (°C), average daily wind speed (m s^-1^), maximum 2-minute sustained daily wind speed (m s^-1^) and rainfall amount (mm day^-1^) for three bloom events analyzed at BB3. Bloom event names, dates, and sample sizes are as follows and correspond to panels in figure: A) BB3-A, 2/20/15-3/17/15, n = 25; B) BB3-B, 7/10/15 -7/22/15, n = 13; C) BB3-C, 12/14/16 - 12/28/16, n = 15).

In spring 2015, bloom events were identified at both BB3 and Cayo. The phytoplankton community was dominated by *Aureoumbra lagunensis* during this time (see [16]). An extended bloom event occurred at BB3 from 2/20–3/17 (BB3-A; Fig 5A). Based on the linear model, over half (adjusted r^2^ = 0.56) of the chlorophyll variability during this time can be explained by inverse relationships with average wind speed and temperature (Table 1). On 2/19, leading up to this bloom event, salinity was 43.2, NH_4_^+^ concentration was 0.44 µM, N + N concentration was 0.46 µM, orthophosphate concentration was 0.25 µM, and silicate concentration was 83.9 µM (S1 Table). After the event (on 3/18), orthophosphate and NH_4_^+^ concentrations increased (to 0.52 µM and 0.69 µM, respectively), while N + N and silicate concentrations were slightly lower (0.02 µM and 79.1 µM, respectively) than on 2/19 (S1 Table).

**Table 1:** Additive linear model coefficients for environmental factors explaining chlorophyll during select bloom events at BB3 from 2015–2016 (BB3-A: 2/20/15-3/17/15, n = 25; BB3-B: 7/10/15 -7/22/15, n = 13; BB3-C: 12/14/16 - 12/28/16, n = 15). Overall model p-value and adjusted r^2^ are in the bottom rows for corresponding bloom events.

	BB3-A 2/20/15-3/17/15		BB3-B 7/10/15 -7/22/15		BB3-C 12/14/16 - 12/28/16	
Environmental parameter	coefficient	p-value	coefficient	p-value	coefficient	p-value
Average wind speed (m s^-1^)	-3.64	0.006			3.37	0.002
Average wind speed – 1 day lag (m s^-1^)					3.28	0.002
Maximum wind speed (m s^-1^)			6.68	0.018		
Temperature (°C)	-9.14	<0.001				
Rain amount (mm day^-1^)						
Model adjusted r^2^	0.56		0.36		0.79	
Model p value	<0.001		0.018		<0.001	

**Fig 6:**
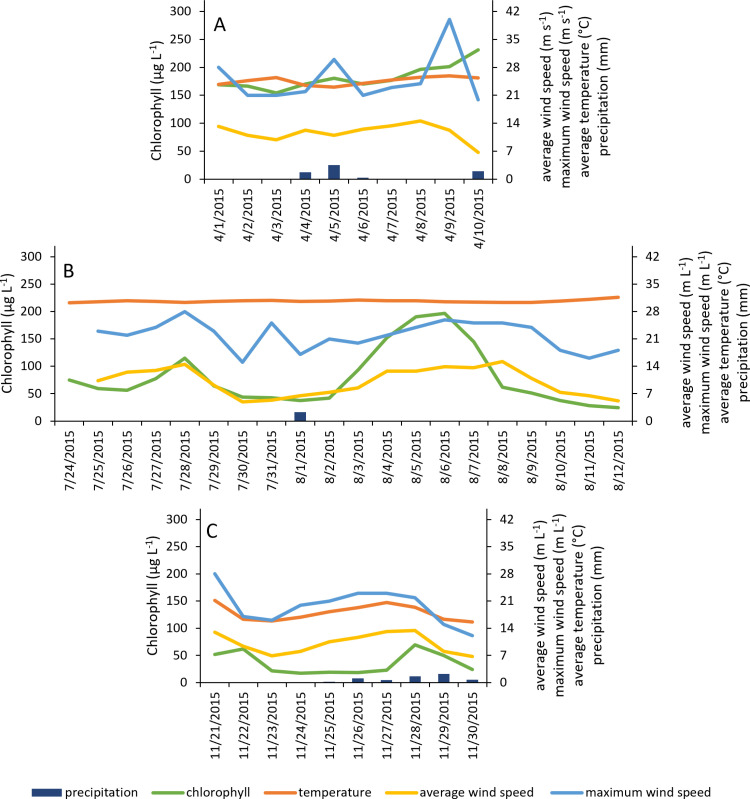
Chlorophyll (µg L^-1^), temperature (°C), average daily wind speed (m s^-1^), maximum 2-minute sustained daily wind speed (m s^-1^) and rainfall amount (mm day^-1^) for three bloom events analyzed at Cayo. Bloom event names, dates, and sample sizes are as follows, and correspond to panels in figure: A) Cayo-A, 4/1/15 - 4/10/15, n = 10; B) Cayo-B, 7/24/15 - 8/12/15, n = 20; C) Cayo-C, 11/21/15 - 11/30/15, n = 10.

Later in spring 2015, there was a bloom at Cayo from 4/1–4/10 (Cayo-A; Fig 6A). Sonde coverage did not capture the decline of the bloom. In contrast to the spring bloom at BB3, temperature was positively related to chlorophyll and, along with rain, explained almost half of the variability in chlorophyll (adjusted r^2^ = 0.45; Table 2). Salinities at both of the adjacent sites decreased from before (3/18) to after (4/16) the bloom event, though the decrease at BB1 was more pronounced (S2 and S3 Tables). The phytoplankton community biovolume at both nearby sites decreased from 3/18–4/16 (see [16]). *A. lagunensis* dominated community biovolume at both sites on both dates, but at BB1 there was a noticeable increase in diatom and dinoflagellate biovolume from 3/18–4/16 (see [16]). NH_4_^+^, N + N, and orthophosphate concentrations at BB1 increased by orders of magnitude from 3/18–4/16 (NH_4_^+^: 0.78 µ M to 2.30 µ M; N + N: 0.01 µ M to 15.92 µ M; orthophosphate: 0.81 µ M to 9.57 µ M), while concentrations at BB3 remained low (S2 and S3 Tables).

**Table 2:** Additive linear model coefficients for environmental factors explaining chlorophyll during select bloom events at Cayo from 2015–2016 (Cayo-A: 4/1/15 - 4/10/15, n = 10; Cayo-B: 7/24/15 - 8/12/15, n = 20; Cayo-C: 11/21/15 - 11/30/15, n = 10). Overall model p-values and adjusted r^2^ are in the bottom rows for corresponding bloom events.

	Cayo-A 4/1/15 - 4/10/15		Cayo-B 7/24/15 - 8/12/15		Cayo-C 11/21/15 - 11/30/15	
Environmental parameter	coefficient	p-value	coefficient	p-value	coefficient	p-value
Average wind speed (m s^-1^)			10.54	0.001		
Average wind speed – 1 day lag (m s^-1^)					6.73	0.008
Maximum wind speed (m s^-1^)						
Temperature (°C)	19.55	0.024				
Rain amount (mm day^-1^)	13.90	0.037				
Model adjusted r^2^	0.45		0.43		0.61	
Model p value	0.050		0.001		0.008	

A summer 2015 bloom event at BB3 (BB3-B) occurred from 7/10–7/22 (Fig 5B). Sonde coverage did not capture the bloom decline. Maximum wind speed was positively correlated with chlorophyll during this event, explaining 36% of the variation in chlorophyll (Table 1). Temperature was invariant throughout the bloom (Fig 5), while salinity increased from 11.7 before to 17.8 after the bloom (6/24–7/23; S1 Table). Silicate concentration decreased sharply, from 68.5 µM to 11.6 µM (S1 Table). N + N and orthophosphate concentrations also decreased, while NH_4_+ concentration increased (5.79 µM to 12.41 µM; S1 Table). In contrast to the spring, the phytoplankton community was more diverse during summer 2015, with diatoms accounting for the largest fraction of community biovolume at BB1 and BB3 (see [16]).

A summer 2015 bloom event at Cayo (Cayo-B) occurred from 7/24–8/12 (Fig 6B). Based on the linear model, there was a significant positive relationship between chlorophyll and average wind speed, with an adjusted r^2^ of 0.43 (Table 2). Temperature remained constant for a large portion of the bloom, with only a slight increase in temperature noted in early August. Salinity increased from before (7/23) to after (8/13) the bloom at both BB1 (15.9 to 20.5) and BB3 (17.8 to 20.4; S2 and S3 Tables). NH_4_+ and N + N concentrations decreased over that time period at both BB1 (NH_4_+: 2.40 µM to 0.61 µM; N + N: 0.65 µM to 0.19 µM) and BB3 (NH_4_+: 12.41 µM to 1.62 µM; N + N: 5.07 µM to 0.27 µM), while silicate concentrations increased at both sites (BB1: 15.9 µM to 27.4 µM; BB3: 11.6 µM to 21.5 µM; S2 and S3 Tables). Orthophosphate concentrations varied little before and after the bloom (BB1: 0.44 to 0.26 µM; BB3: 0.23 µM to 0.36 µM; S2 and S3 Tables). Phytoplankton community biovolume at BB1 and BB3 before (7/23) and after (8/13) the bloom event was dominated by benthic and planktonic diatoms, as well as *A. lagunensis* (see [16]).

Winter blooms were observed at both BB3 and Cayo, though in different years. The Cayo winter bloom was observed from 11/21/15–11/30/15 (Cayo-C; Fig 6C). Based on cell count data from BB1 and BB3 on 11/19 and phytoplankton live screens from 12/10, the community during this bloom consisted of a mix of dinoflagellates, *Mesodinium rubrum*, *A. lagunensis*, and planktonic, chain-forming diatoms (see [16]). During this bloom, chlorophyll was positively correlated with one-day lagged average wind speed, explaining 61% of the variation in chlorophyll (Table 2). From before (11/15) to after (12/10) the bloom event, there were decreases in orthophosphate and silicate concentrations at both BB1 and BB3 (S2 and S3 Tables). The decrease in silicate was sharp, from 81.3 µM to 9.1 µM at BB1, and from 89.1 µM to 0.4 µM at BB3 (S2 and S3 Tables). DIN concentrations increased at BB1 (NH_4_+: 3.59 µM to 7.49 µM; N + N: 0.35 µM to 0.53 µM) and decreased at BB3 (NH_4_+: 14.49 µM to 3.46 µM; N + N: 1.12 µM to 0.59 µM; S2 and S3 Tables).

A winter bloom was observed at BB3 from 12/14/16–12/28/16 (BB3-C; Fig 5C). Although cell counts were not available from this time, phytoplankton live screens indicated a mix of diatoms, dinoflagellates, and small flagellates. From the linear model, both average wind speed and one-day lagged average wind speed were significantly related to chlorophyll, together explaining 79% of variability in chlorophyll during the event (Table 1). On 12/14, at the beginning of this bloom event, salinity was 43.2, silicate was high (139.3 µM), and NH_4_+ and N + N were 8.65 µM and 3.73 µM, respectively (S1 Table). Temperature did fluctuate throughout the bloom event (Fig 5), though it did not correlate to chlorophyll (Table 1). No post-bloom environmental data from January 2017 is available, as sampling was prevented due to poor weather.

### Effects of different sampling frequencies on simulated annual chlorophyll concentrations

Chlorophyll concentrations at BB3 averaged 69.7 µg L^-1^ and ranged from 8.2 µg L^-1^ to 305.9 µg L^-1^ in 2015 (n = 171; Fig 2). The distribution of chlorophyll measurements was skewed right; while the majority of measurements were < 40 µg L^-1^, some reached > 300 µg L^-1^. Measurements > 100 µg L^-1^ were all from the spring 2015. The average of the annual chlorophyll concentration calculated from the monthly (69.5 µg L^-1^) and quarterly (69.0 µg L^-1^) simulations did not differ from that of the raw dataset (69.7 µg L^-1^; Table 3). However, with simulated quarterly sampling, the maximum concentration reported for each simulated year was, on average, 174.6 µg L^-1^, ~ 131 µg L^-1^ less than the raw data maximum (Table 3). The minimum concentration captured with quarterly sampling was, on average, 24.9 µg L^-1^, ~17 µg L^-1^ higher than the raw data minimum. With simulated monthly sampling, the maximum concentration averaged 260.4 µg L^-1^ (~ 46 µg L^-1^ lower than raw data maximum) and the minimum concentration averaged 17.2 µg L^-1^ (~9 µg L^-1^ higher than the raw data minimum; Table 3).

**Table 3:** Descriptive statistics for chlorophyll measurements during the simulated year of sampling, for quarterly and monthly sampling simulations.

	Quarterly sampling (simulated)	Monthly sampling (simulated)	Raw data (not simulated)
Average annual mean chlorophyll (µg L^-1^)	69.0	69.5	69.7
Average chlorophyll minimum (µg L^-1^)	24.9	17.2	8.2
Average chlorophyll maximum (µg L^-1^)	174.6	260.4	305.6

## Discussion

In this study we combined two years of high-frequency water quality data collections with data from a monthly sampling program to investigate timescales of chlorophyll variability and drivers of blooms in Baffin Bay, Texas. Results show that event-scale (i.e., sub-monthly) chlorophyll variability was higher than variability associated with lower frequency timescales (i.e., seasonal, interannual), a common phenomenon in coastal systems [2]. We were also able to elucidate drivers of short-term variability in phytoplankton population dynamics, which is sorely lacking because many sampling programs are unable to sample at high frequencies due to monetary or logistical concerns. Results presented expand upon the currently limited knowledge of short-term chlorophyll variability in long residence time, low inflow estuarine systems. Findings also offer insight into design considerations for phytoplankton/water quality monitoring programs in similar estuarine systems.

### Timescales of chlorophyll variability (2014–2018)

The magnitude of high frequency (sub-monthly) variability was higher than that of monthly and interannual variability in Baffin Bay. High frequency variability in phytoplankton biomass is common in coastal systems [2] and may reflect factors affecting phytoplankton growth or biomass distribution that occur on timescales of hours-days (e.g., wind, tides, advection; [6,17]), as well as episodic changes in phytoplankton biomass due to ephemeral rain events [4,7,18]. As discussed later, the sub-monthly variability in Baffin Bay likely reflects the relative importance of short-timescale processes such as wind and localized, ephemeral rain events [1,18,19]. In nearby San Antonio Bay [18] and in other systems worldwide (e.g., [1,17]), wind was implicated as an important driver of short-term chlorophyll variability.

Based on a similar analysis conducted by Cloern and Jassby [2] on coastal systems worldwide, in which the median sub-monthly variability among 84 datasets was found to be 0.59, the sub-monthly variability in Baffin Bay is relatively low (0.37). This may be explained in part by the absence of a tidal influence in Baffin Bay, in contrast to estuaries such as the Matanzas River Estuary (Florida; [20]) or North Inlet (South Carolina; [6]) where tides have a strong influence on phytoplankton biomass. It may also be explained by the long-residence time of Baffin Bay, which would allow for extended effects of high rainfall periods, in contrast to systems with rapid flushing that would return conditions to baseline quickly (Rhode River subestuary, Chesapeake Bay; St. Lucie Estuary, Florida; [21,22]).

While not as large in magnitude as sub-monthly variability, there was also significant variability in chlorophyll at interannual timescales in Baffin Bay. During 2014–2018, effects of both non-El Niño (drought) and El Niño (high rainfall) conditions were observed and are a primary cause of the high interannual phytoplankton variability in chlorophyll [16,23]. Climatic phenomena such as the Pacific Decadal Oscillation and North Atlantic Oscillation have been associated with interannual variability in chlorophyll in other subtropical and temperate coastal systems [1,2,24]. In Texas, El Niño conditions lead to high rainfall and low salinities in the coastal zone [25]. The periodicity of ENSO along the Texas coast is estimated to be 4–5 years [25], so this interannual variability can be expected to have a recurring imprint on phytoplankton dynamics in Baffin Bay and other Texas estuaries.

Seasonal timescales contributed the least to variability in chlorophyll in Baffin Bay. In the Patos Lagoon Estuary, Brazil, another subtropical, microtidal lagoon, the lack of a consistent bloom season was thought to result in a depressed seasonal pattern [1,2]. In contrast, in many temperate river-dominated estuarine systems phytoplankton dynamics exhibit pronounced seasonal trends (e.g., Chesapeake Bay; [26]). Overall, the distinction between subtropical systems like Baffin Bay and temperate systems is consistent with the positive relationship between seasonal variability and latitude demonstrated by Cloern and Jassby [2].

### Potential drivers of phytoplankton blooms

Bloom events were identified across seasons and years at both sites, corresponding with a range of temperature and salinity conditions. Wind-related variables (average speed, maximum speed, one-day lagged average wind speed) were significant factors related to chlorophyll in five of the six bloom events assessed, indicating that wind is an important driver of phytoplankton dynamics in Baffin Bay. High wind may inject nutrients from sediment porewater into the water column while also resuspending sediments that may release nutrients [27,28], stimulating phytoplankton growth in surface waters [29–31]. High wind may also resuspend phytoplankton cells, directly increasing phytoplankton biomass in the surface waters [32–34]. Wind has been linked to short-term chlorophyll variability in nearby San Antonio Bay [18] and in other systems worldwide [1,17].

During the summer blooms, wind was the only factor related to chlorophyll, and wind alone predicted ~40% of chlorophyll variability during both events. Summer tends to be a dry period in South Texas, with relatively little rainfall and high rates of evaporation [35,36]. These conditions were reflected by salinity increases at BB1 and BB3 over the course of both bloom events and by the complete absence of rain during one event. Without external nutrient inputs during the summer months, wind-driven resuspension of nutrients may be an important source of nutrient flux into the surface water. The increase in NH_4_^+^ from before to after the bloom event at BB3 reflects a wind-driven release of NH_4_^+^ from sediments [27], while the simultaneous decrease in silicate is consistent with uptake by diatoms, which may have been resuspended by wind. The surface water diatom community at BB3 during July and August 2015 was primarily benthic taxa (Naviculoids and *Cylindrotheca*; Cira unpubl. data), supporting this interpretation of the data. While the bloom decline was not captured in the BB3 bloom event, it was captured during the Cayo bloom. The positive correlation between wind and chlorophyll for the duration of the bloom indicates that not only was high wind associated with bloom initiation, but also that relaxation of wind was associated with bloom decline. This could either be due to a cessation of nutrient flux or sedimentation of diatoms cells [34]. On the two days following peak chlorophyll, concentrations decreased by 26% and 57% each day. Based on sinking velocities described by Durante et al. [37] for simple, elongated cells, the sinking rate of the dominant benthic diatom group enumerated (Naviculoids of length ~ 30 µm) would be ~ 0.6 m d^-1^. Thus, these cells could be completely removed from the surface water within one day. Additionally, biological processes like grazing and benthic filter feeders were not explored here but are known to play a role in short-term phytoplankton dynamics [4,38], particularly in summer months, and should be considered in future studies addressing the decline of blooms in this system.

As during summer, there was also a significant relationship between chlorophyll and wind during winter. A key difference however was that there was a one-day time lag between wind speed increases and chlorophyll increases during the winter months. We suspect that this is due to the slower phytoplankton growth rates seen in colder months [3,4]. The presence of a time lag also suggests that the increase in chlorophyll is due to phytoplankton growth rather than merely resuspension of phytoplankton cells. Benthic phytoplankton biomass tends to be lower in winter months than during spring or summer [18], suggesting that overall, wind-driven resuspension of phytoplankton cells may be less important in winter months compared with other times of year. Furthermore, the plankton community during this time consisted primarily of planktonic (centric) rather than benthic (pennate) diatom taxa.

In the spring of 2015, blooms occurred at BB3 and Cayo despite negative (BB3) or non-existent (Cayo) relationships between wind and chlorophyll. One aspect of these blooms that was different from the aforementioned summer and winter blooms was the high abundance of *A. lagunensis*. For the Cayo spring bloom, chlorophyll was positively correlated with temperature and rain. There are two possibilities behind the positive relationship between temperature and chlorophyll, and they are not mutually exclusive. The first is simply that phytoplankton growth tends to increase with increasing temperatures [39], which (temperature increase) in this case would have also corresponded to increasing daylength and light availability. The combination of these factors has long been known to lead to classic spring blooms in oceanic systems [8]. Another possibility is that the increased temperature stimulated nutrient regeneration, which is temperature-dependent [40]. Indeed, DIN concentrations were low prior to the bloom, and N:P ratios were indicative of N-limiting conditions. Thus, factors increasing N-availability would likely have had a positive impact on phytoplankton growth. This may also explain the positive relationship between chlorophyll and rain, which would have supplied additional nutrients to the system. In contrast to the spring bloom event at Cayo, chlorophyll during the spring bloom event at BB3 was negatively correlated with both wind and temperature. Late winter/spring is characterized by the passage of northerly fronts in ~ 5 d intervals, each typically associated with wind speeds > 15 m s^-1^, an abrupt shift in wind direction from the southeast to the north, and often a drop in air temperature [41–43]. The environmental variability at this time, combined with the lag in phytoplankton response identified during the winter blooms (though not explicitly identified in the spring), complicates our ability to link these factors directly with a biologic response. However, instances of wind speeds > 15 m s^-1^ were followed by an increase in chlorophyll twice throughout the BB3 spring bloom event, suggesting chlorophyll increases after passage of northerly fronts, potentially resulting in a negative correlation with wind. Springtime blooms in other systems have been associated with periods of strong wind followed by a relaxation of wind [44]. MacIntyre et al. [45] describe high wind events as triggering formation of *Aureococcus anophagefferens* blooms by creating conditions favorable for its growth (high turbidity/low light, high organic nutrients). Because of its slow growth rate, bloom formation after these conditions developed would not be expected to be immediate. A similar relationship is expected to exist for *A. lagunensis*, because it is adapted to the high turbidity/low light conditions that may be caused by wind [46,47], can utilize organic nitrogen forms (e.g., [48]), and has relatively slow growth [46]. Similar to wind, the relationship with temperature may be an artifact of the passage of cold fronts in the winter/spring months, as colder air moves in after the passage of the front; a sharp temperature drop was associated with the first increase in chlorophyll during this bloom event, which also corresponded to a relaxation of wind speed. Further research is needed to identify the primary drivers of chlorophyll variability during this time of highly variable environmental conditions.

While the analysis of high frequency data here highlights the importance of wind to short-term phytoplankton dynamics in Baffin Bay, it is important to reiterate that many other factors such as nutrient concentrations and grazing pressure have been shown to play significant roles in bloom dynamics. Short-term nutrient addition bioassays conducted at a similar time as this analysis (2015) demonstrate a stimulatory effect of nitrogen on phytoplankton growth in Baffin Bay, suggesting that nitrogen concentration is an important driver of phytoplankton dynamics in the bay on daily timescales [49]. Additionally, on longer timescales, a disruption in grazing pressure is thought to have contributed to a previous bloom in the system [50]. These factors are more onerous to assess on hourly-daily timescales, but knowledge of them would be needed to comprehensively assess drivers of bloom dynamics in the system.

### Effects of sampling frequency

Monthly and quarterly sampling are common frequencies for water quality monitoring programs in the U.S. and elsewhere, and both frequencies have been used to sample Baffin Bay. Most bloom events identified with the high-frequency sampling in this study lasted less than three days, so these blooms would likely not be captured by traditional (monthly, quarterly) sampling approaches. This effect was seen in sampling simulations as a reduction in the maximum chlorophyll concentration captured with either monthly or quarterly sampling approaches. Compared with the raw dataset, the maximum chlorophyll concentration was reduced by 15% in monthly sampling simulations and by 43% in quarterly sampling simulations. Similar relationships between sampling frequencies and chlorophyll variability have been reported elsewhere (Baltic Sea, [51]), highlighting the limitations of traditional sampling frequencies in capturing short-lived phytoplankton blooms.

The appropriate sampling resolution depends on chlorophyll variability in the system [51]. As noted here, inadequate sampling frequency may cause blooms to be missed and lead to an incomplete/inaccurate understanding of drivers of phytoplankton dynamics [1,51]. However, depending on available resources and research goals, sampling at a high frequency may not always be recommended. In short, sampling frequency should be aligned with research needs. If the specific goal is to capture bloom events that develop and decline on timescales of days, monthly or quarterly sampling is clearly inadequate, and daily resolution sampling may be needed. However, if the goal is to describe interannual or long-term trends, then monthly scales may be a preferred frequency because, as shown here, they may adequately capture the annual mean chlorophyll without the need for more labor intensive, costlier higher frequency sampling. As done in this study, a combination of monitoring methods may be an acceptable approach to balance data needs and resource limitations.

## Conclusions

As demonstrated here, high frequency chlorophyll variability operating on the sub-monthly timescale was important and is not captured by low frequency monitoring programs. Results showed that while the average chlorophyll concentration was reasonably well approximated even with lower frequency sampling, short-lived blooms would clearly be missed. This study identified wind is an important driver of phytoplankton population dynamics on daily time scales in Baffin Bay. This is important locally given that wind speeds along the Texas coast are projected to increase due to climate change [52,53], which suggests that short-duration blooms may become more common in the ecosystem in the future. The high frequency variability seen here represents a large portion of chlorophyll variability in many systems worldwide [2,3,54,55]. Unfortunately, most sampling programs do not operate at these high frequency timescales and consequently relatively little is still known about the role of short-term processes on estuarine phytoplankton ecology. Nonetheless, it is important to acknowledge that drivers of phytoplankton variability occur on a continuum of scales [12], and quantification of scales of variability will help distinguish phytoplankton responses to short-term drivers from those attributable to long-term, lower-frequency factors like climate change and eutrophication [56,57].

## Supporting information

S1 Fig Raw dataset “year” (n = 171) used in the resampling simulations, color-coded based on the month (M) or quarter (Q) each datapoint was grouped in for the resampling simulations. See Figure 2A for spread of 171 chlorophyll datapoints in 2015.
(TIF)

S1 Table Data from monthly water quality monitoring samples collected at BB3 were used to describe environmental conditions before and after the bloom events BB3-A, BB3-B, and BB3-C. All parameters reported (including temperature and salinity) are from monthly monitoring data. No post-BB3-C data is reported because sampling was not conducted in January 2017.
(DOCX)

S2 Table Data from monthly water quality monitoring samples collected at BB3 were used to describe e environmental conditions before and after the bloom events Cayo-A, Cayo-B, and Cayo-C. All parameters reported (including temperature and salinity) are from monthly monitoring data.
(DOCX)

S3 Table Environmental conditions before and after the bloom events Cayo-A, Cayo-B, and Cayo-C, from monthly water quality monitoring samples collected at BB1. All parameters reported (including temperature and salinity) are from monitoring data.
(DOCX)
