# Supplementary figures and images for: Timescales and drivers of chlorophyll variability in a subtropical, long residence time estuary (Baffin Bay, Texas, USA)
Source: PLoS One. 2025 May 9;20(5):e0322053. doi: 10.1371/journal.pone.0322053 (PMC12063824; doi:10.1371/journal.pone.0322053)

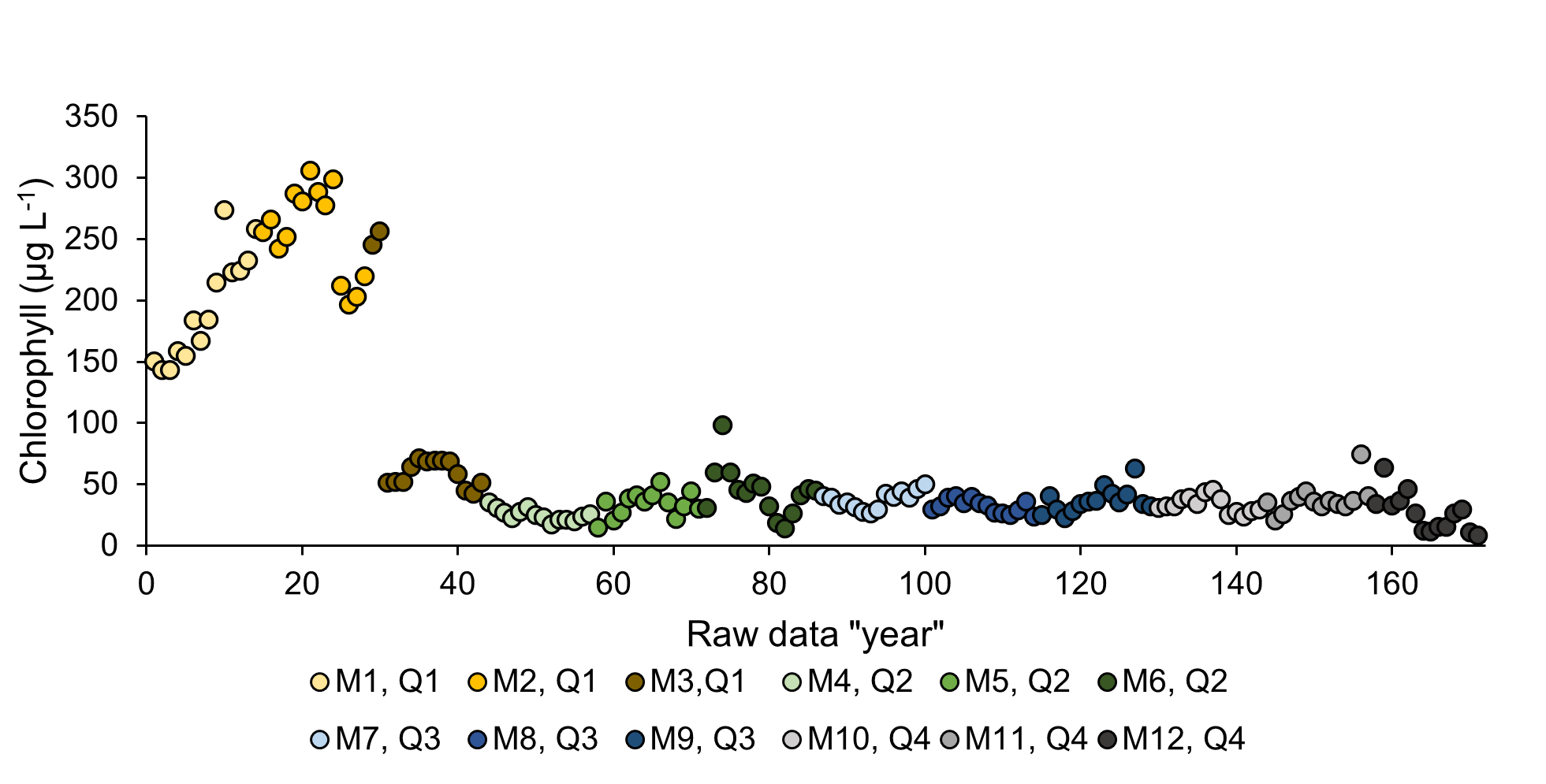

Supplement: S1 Fig — See Figure 2A for spread of 171 chlorophyll datapoints in 2015. (TIF) [file pone.0322053.s001.tif]
