# Supplementary material for: Timescales and drivers of chlorophyll variability in a subtropical, long residence time estuary (Baffin Bay, Texas, USA)
Source: PLoS One. 2025 May 9;20(5):e0322053. doi: 10.1371/journal.pone.0322053 (PMC12063824; doi:10.1371/journal.pone.0322053)
Supplement: S1 Table — All parameters reported (including temperature and salinity) are from monthly monitoring data. No post-BB3-C data is reported because sampling was not conducted in January 2017. (DOCX) [file pone.0322053.s002.docx]

Supplemental Table 1. Environmental conditions before and after the bloom events BB3-A, BB3-B, and BB3-C, from monthly water quality monitoring samples collected at BB3. All parameters reported (including temperature and salinity) are from monitoring data. No post-BB3-C data is reported because sampling was not conducted in January 2017.

|  | **Pre –**  **BB3-A** | **Post –**  **BB3-A** | **Pre –**  **BB3-B** | **Post –**  **BB3-B** | **Pre –**  **BB3-C** |
| --- | --- | --- | --- | --- | --- |
| **Date** | **2/19/2015** | **3/18/2015** | **6/24/2015** | **7/23/2015** | **12/14/2016** |
| Temperature (°C) | 14.2 | 19.6 | 28 | 29 | 17.3 |
| Salinity | 43.2 | 40.1 | 11.7 | 17.8 | 43.2 |
| NH_4_^+^ (µM) | 0.44 | 0.69 | 5.79 | 12.41 | 8.65 |
| N+N (µM) | 0.46 | 0.02 | 5.58 | 5.07 | 3.73 |
| DON (µM) | 58.1 | 55.1 | 41.9 | 67.7 | 97.8 |
| Silicate (µM) | 83.9 | 79.1 | 68.5 | 11.6 | 139.3 |
| DOC (µM) | 1019 | 845 | 647 | 605 | 1121 |
| Orthophosphate (µM) | 0.25 | 0.52 | 0.69 | 0.23 | 0.42 |
