# Supplementary material for: Timescales and drivers of chlorophyll variability in a subtropical, long residence time estuary (Baffin Bay, Texas, USA)
Source: PLoS One. 2025 May 9;20(5):e0322053. doi: 10.1371/journal.pone.0322053 (PMC12063824; doi:10.1371/journal.pone.0322053)
Supplement: S2 Table — All parameters reported (including temperature and salinity) are from monthly monitoring data. (DOCX) [file pone.0322053.s003.docx]

Supplemental Table 2. Environmental conditions before and after the bloom events Cayo-A, Cayo-B, and Cayo-C, from monthly water quality monitoring samples collected at BB3. All parameters reported (including temperature and salinity) are from monitoring data.

|  | **Pre – Cayo-A** | **Post – Cayo-A** | **Pre – Cayo-B** | **Post – Cayo-B** | **Pre – Cayo-C** | **Post – Cayo-C** |
| --- | --- | --- | --- | --- | --- | --- |
| **Date** | **3/18/2015** | **4/16/2015** | **7/23/2015** | **8/13/2015** | **11/19/2015** | **12/10/2015** |
| Temperature (°C) | 19.6 | 23.5 | 29 | 30.3 | 20.7 | 18.5 |
| Salinity | 40.1 | 34.6 | 17.8 | 20.4 | 30.9 | 31.5 |
| NH_4_^+^ (µM) | 0.69 | 1.14 | 12.41 | 1.62 | 14.49 | 3.46 |
| N+N (µM) | 0.02 | 0.16 | 5.07 | 0.27 | 1.12 | 0.59 |
| DON (µM) | 55.1 | 57.8 | 67.7 | 55.6 | 72.0 | 73.0 |
| Silicate (µM) | 79.1 | 93.4 | 11.6 | 21.5 | 89.1 | 0.4 |
| DOC (µM) | 845 | 919 | 605 | 712 | 867 | 896 |
| Orthophosphate (µM) | 0.52 | 0.28 | 0.23 | 0.36 | 0.57 | 0.23 |
