# Supplementary material for: Timescales and drivers of chlorophyll variability in a subtropical, long residence time estuary (Baffin Bay, Texas, USA)
Source: PLoS One. 2025 May 9;20(5):e0322053. doi: 10.1371/journal.pone.0322053 (PMC12063824; doi:10.1371/journal.pone.0322053)
Supplement: S3 Table — All parameters reported (including temperature and salinity) are from monitoring data. (DOCX) [file pone.0322053.s004.docx]

Supplemental Table 3. Environmental conditions before and after the bloom events Cayo-A, Cayo-B, and Cayo-C, from monthly water quality monitoring samples collected at BB1. All parameters reported (including temperature and salinity) are from monitoring data.

|  | **Pre – Cayo-A** | **Post – Cayo-A** | **Pre – Cayo-B** | **Post – Cayo-B** | **Pre – Cayo-C** | **Post – Cayo-C** |
| --- | --- | --- | --- | --- | --- | --- |
| **Date** | **3/18/2015** | **4/16/2015** | **7/23/2015** | **8/13/2015** | **11/19/2015** | **12/10/2015** |
| Temperature (°C) | 21.8 | 22.7 | 28.4 | 29.3 | 19.9 | 19.8 |
| Salinity | 32.5 | 13.0 | 15.9 | 20.5 | 28.0 | 29.3 |
| NH_4_^+^ (µM) | 0.78 | 2.30 | 2.40 | 0.61 | 3.59 | 7.49 |
| N+N (µM) | 0.01 | 15.92 | 0.65 | 0.19 | 0.35 | 0.53 |
| DON (µM) | 62.2 | 51.7 | 60.0 | 68.2 | 69.6 | 73.6 |
| Silicate (µM) | 75.1 | 106.7 | 15.9 | 27.4 | 81.3 | 9.1 |
| DOC (µM) | 879 | 739 | 686 | 792 | 839 | 844 |
| Orthophosphate (µM) | 0.81 | 9.57 | 0.44 | 0.26 | 1.64 | 1.56 |
